# Prevalence of Vitamin D Deficiency among Patients of Acute Coronary Syndrome in a Tertiary Care Center of Eastern Nepal

**DOI:** 10.31729/jnma.5166

**Published:** 2021-03-31

**Authors:** Richa Nepal, Prahlad Karki, Surendra Uraw, Madhab Lamsal

**Affiliations:** 1 Department of Internal Medicine, B.P. Koirala Institute of Health Sciences, Dharan, Nepal; 2 Department of Biochemistry, B.P. Koirala Institute of Health Sciences, Dharan, Nepal

**Keywords:** *acute coronary syndrome*, *myocardial infarction*, *prevalence*, *vitamin D*

## Abstract

**Introduction:**

Vitamin D deficiency is an emerging risk factor for cardiovascular diseases. Very few studies have been done to find out vitamin D deficiency status among cardiovascular patients in Nepalese setup. This research aims to find out the prevalence of vitamin D deficiency among patients of acute coronary syndrome admitted in a tertiary care center of eastern Nepal.

**Methods:**

This was a descriptive cross-sectional study conducted among patients of acute coronary syndrome admitted in a tertiary care hospital from 1st February 2018 to 31st July 2018. Ethical clearence was taken from Institutional Review Committee of B.P. Koirala Institute of Health Sciences (Reference number: 259/074/075-IRC). Convenience sampling method was used. Data was entered in Microsoft Excel and analyzed using Statistical Package for the Social Sciences version 25. Point estimate at 95% Confidence Interval was calculated along with frequency and proportion for binary data.

**Results:**

A total of 33 (64.7%) at 95% Confidence Interval (51.58-77.82) patients of acute coronary syndrome had vitamin D deficiency in our study with 19 (37.3%) having mild deficiency and 14 (27.4%) having moderate deficiency. None of the patients had severe vitamin D deficiency in our study. The mean vitamin D levels were lower in diabetics (23.57±9.28ng/ml) as compared to nondiabetics (31.91±12.50ng/ml), in hypertensive patients (24.36±7.67ng/ml) as compared to nonhypertensive patients (30.97±13.72ng/ml), and in patients with dyslipidemia (22.86±6.44ng/ml) as compared to those without dyslipidemia (37.68±13.15ng/ml).

**Conclusions:**

Prevalence of vitamin D deficiency among patients of acute coronary syndrome in our study was comparable to various other homologous international studies.

## INTRODUCTION

A growing body of evidence has linked inadequate vitamin D levels to major non-skeletal diseases, especially cardiovascular diseases.^1^ Acute coronary syndrome (ACS) is one of the leading cardiovascular diseases, to cause significant morbidity and mortality in patients. It has been associated with various modifiable and non-modifiable risk factors, out of which vitamin D deficiency is the novel risk factor with potential therapeutic implications.^2^

Observational studies have associated chronic vitamin D deficiency to classical cardiovascular risk factors like hypertension, diabetes mellitus, dyslipidemia and metabolic syndrome.^3^ The prevalence of vitamin D deficiency ranged from 80 to 95% in patients of coronary artery disease in studies, done in different parts of the world.^4-5^ Data related to vitamin D deficiency, in patients with cardiovascular diseases, is very limited in Nepalese setup.

This research aims to find out the prevalence of vitamin D deficiency among patients of acute coronary syndrome,admitted in a tertiary care center of eastern Nepal.

## METHODS

This was a descriptive cross-sectional study conducted over a span of six months from 1^st^ February, 2018 till 31^st^ July, 2018. The study was conducted in B.P. Koirala Institute of Health Sciences (BPKIHS) which is the tertiary care center located in eastern Nepal. The Institutional Review Committee (IRC) of BPKIHS had approved our protocol prior to starting the study (reference number: 259/074/075-IRC). All the consecutive patients, presenting to BPKIHS emergency during the study period, who were diagnosed to have acute coronary syndrome, were included in this study. Patients who were on vitamin D supplement or on any other medications, which interfered with vitamin D levels, were excluded from this study. These medications included glucocorticoids, anti-epileptics like phenytoin, phenobarbital, and carbamazepine, antiretroviral drugs and anti-tubercular drugs like rifampicin. Similarly, patients of chronic kidney disease, chronic liver disease and parathyroid related endocrine disorders were also excluded. Patients were included in this study, only after they gave written consent for history taking, physical examination, and necessary investigations. All the consecutive patients of acute coronary syndrome were included in this study after the inclusion and exclusion criteria were taken into account. The sample size was calculated using the formula,

$$\begin{array}{ll} \text{n} & ={\text{Z}}^{\text{2}}\times \text{p}\times \text{q}/{\text{e}}^{\text{2}}\\ & ={\left(1.645\right)}^{2}\times (0.835)\times (1-0.835)/{\left(0.09\right)}^{\text{2}}\\ & =46 \end{array}$$

Where,

n = required sample size,
Z = 1.645 at 90% Confidence Interval
p = prevalence of vitamin D deficiency among patients of ACS in reference population, 83.5%^4^
q = 1-p
e = margin of error, 9%

Hence, the calculated sample size was 46. Taking a 10% non-response rate, we included 51 participants in the study.

Data was collected on a structured questionnaire, which included questions on demographic variables and relevant clinical history. Detailed clinical examination was done and diagnosis of acute coronary syndrome was made based on clinical history, ECG findings and raised levels of cardiac biomarkers during admission. Cases of unstable angina, non-ST elevated myocardial infarction, and ST elevated myocardial infarction were all included. Blood samples for relevant baseline investigations were collected at the time of admission. Sample for lipid profile and vitamin D was drawn after eight hours of fasting on the next day of admission from BPKIHS ward and coronary care unit. Patients were diagnosed to have dyslipidemia as per National Cholesterol Education Program Adult Treatment Panel (NCEP ATP III) guidelines. Following clinical stability, as the patients were allowed to be mobilized, body mass index (BMI) was calculated.

Laboratory estimation of vitamin D level was done on Maglumi fully-autochemiluminescence immunoassay (CLIA) analyzer in the Department of Biochemistry of BPKIHS. Two milliliters of blood was centrifuged at room temperature. The collected serum was incubated twice, after which the washed sample underwent chemiluminescent reaction to measure relative light units (RLU) that gave the measure of 25-OH-vitamin D in serum. Vitamin D level of 20-29ng/ml was considered as insufficiency (also termed as mild deficiency), 10 to 19ng/ml was considered as moderate deficiency, and less than 10ng/ml was considered as severe deficiency, according to BPKIHS biochemistry laboratory values.

Data was entered in an excel sheet and analyzed by IBM Statistical Package for the Social Sciences version 25. Descriptive statistics were presented with frequencies and percentages for categorical variables, and mean along with standard deviation for continuous variables.

## RESULTS

**Figure 1.**
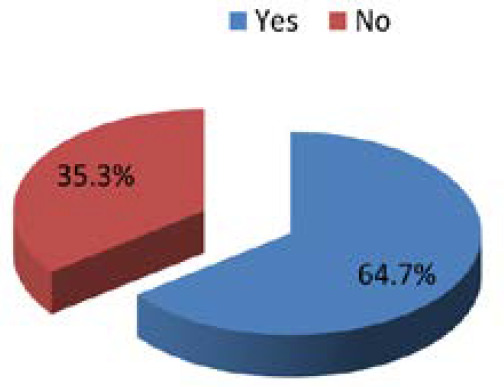
Deficiency of Vitamin D in the patients of ACS.

A total of 51 patients of acute coronary syndrome were enrolled in this study out of which 33 (64.7%) (51.58-77.82 at 95% CI) patients of acute coronary syndrome had vitamin D deficiency (Figure 1).

Out of the vitamin D deficient patients, 19 (37.3%) had mild deficiency and 14 (27.4%) had moderate deficiency (Table 1).

**Table 1:** Vitamin D levels in the patients of ACS.

Vitamin D levels	Frequency n (%)
10ng/ml to 19ng/ml (Moderate deficiency)	14 (27.4)
20ng/ml to 29ng/ml (Mild deficiency)	19 (37.3)
30ng/ml and more (Normal value)	18 (35.3)

The mean age of the patients was 61.7±11.70 years. Out of total patients, 33 (64.7%) were of age 60 years and above, and 18 (35.3%) were females. Around 41 (80%) patients were from terai region of Nepal, 17 (33%) patients consumed vegetarian diet and 24 (47%) of them were engaged in occupation that involved outdoor activities. Twenty-four (47%) of them were diabetic, 23 (45%) were hypertensive, 20 (39%) were obese with body mass index of ≥25 kg/m^2^, and 32 (62.7%) were smokers. Around 33 (64.7%) patients had some form of dyslipidemia on evaluation (Table 2).

**Table 2:** Baseline characteristics of the patients of ACS.

Variables	Category	n (%)
Age	< 60 years	18 (35.3)
	≥ 60 years	33 (64.7)
Gender	Female	18 (35.3)
	Male	33 (64.7)
Address	Terai	41 (80.4)
	Hilly	10 (19.6)
Occupation	Indoors	27 (52.9)
	Outdoors	24 (47.1)
Diabetes mellitus	Yes	24 (47.05)
	No	27 (52.95)
Hypertension	Yes	23 (45.09)
	No	28 (54.91)
Active Smoker	Yes	32 (62.7)
	No	19 (37.3)
	Underweight (<18.5kg/m^2^)	6 (11.76)
Body mass index (BMI)	Normal (18.5-24.9kg/m^2^)	25 (49.01)
	Overweight (25-29.9kg/m2)	15 (29.41)
	Class I Obesity (30-34.5kg/m^2^)	2 (3.92)
	Class 2 Obesity (35-39.9kg/m^2^)	3 (5.88)
Diet	Vegetarian	17 (33.3)
	Mixed	34 (66.7)
Dyslipidemia	Present	33 (64.7)
	Absent	18 (35.3)

According to our lab reference values, 19 (37.3%) patients had mild vitamin D deficiency and 14 (27.4%) patients had moderate vitamin D deficiency. None of the patients had severe vitamin D deficiency in our study. The mean vitamin D level was 27.99±11.77ng/ml with the minimum value of 15.82ng/ml and maximum value of 64.53ng/ml. Mean vitamin D levels and vitamin D deficiency status of the study population, stratified on the basis of baseline characteristics (Table 3). The mean vitamin D levels were found to be lower in females (21.62±6.81 ng/ml) as compared to males (31.46±12.51ng/ml), in patients of age group ≥60 years (25.71±8.75ng/ml) as compared to patients of <60 years (32.17±15.32ng/ml), and in patients from hilly region (22.03±5.37ng/ml) as compared to terai region (29.44±12.48ng/ml) of Nepal. Similarly, the mean vitamin D level was also found to be lower in diabetic (23.57±9.28ng/ml) as compared to non-diabetic patients with ACS (31.91±12.50ng/ml). Hypertensive patients had lower mean vitamin D level (24.36±7.67ng/ml) as compared to non-hypertensive patients (30.97±13.72ng/ml) in our study. Likewise, patients of ACS with dyslipidemia had lower mean vitamin D level (22.86±6.44ng/ml) than those without dyslipidemia (37.68±13.15ng/ml)(Table 3).

**Table 3:** Mean vitamin D levels and vitamin D deficiency status according to the baseline characteristics in the patients of ACS.

Baseline characteristics		Frequency n (%)	Vitamin D (ng/ml)	Vitamin D deficiency status	
	Mean±SD^*^		Yes n (%)	No n (%)
Gender	Female	18 (35.3)	21.62±6.81	16 (31.4)	2 (3.9)
	Male	33 (64.7)	31.46±12.51	17 (33.3)	16 (31.4)
Age	<60 yrs	18 (35.3)	32.17±15.32	10 (19.6)	8 (15.7)
	≥60yrs	33 (64.7)	25.71±8.75	23 (45.1)	10 (19.6)
Address	Terai	41 (80.4)	29.44±12.48	24 (47.1)	17 (33.3)
	Hilly	10 (19.6)	22.03±5.37	9 (17.6)	1 (2.0)
Diet	Vegetarian	17 (33.3)	26.51±10.58	12 (23.5)	5 (9.8)
	Mixed	34 (66.7)	28.73±12.41	21 (41.2)	13 (25.5)
Diabetes Mellitus	Yes	24 (47.1)	23.57±9.28	19 (37.3)	5 (9.8)
	No	27 (52.9)	31.91±12.50	14 (27.5)	13 (25.4)
Hypertension	Yes	23 (45.1)	24.36±7.67	17 (33.3)	6 (11.8)
	No	28 (54.9)	30.97±13.72	16 (31.4)	12 (23.5)
Obesity	Yes	20 (39.2)	25.48±7.64	15 (29.4)	5 (9.8)
	No	31 (60.8)	22.70±6.56	18 (35.3)	13 (25.5)
Dyslipidemia	Yes	33 (64.7)	22.86±6.44	28 (54.9)	5 (9.8)
	No	18 (35.3)	37.68±13.15	5 (9.8)	13 (25.5)
Smoking	Yes	32 (62.7)	29.82±13.50	18 (35.3)	14 (27.4)
	No	19 (37.3)	24.90±7.42	15 (29.4)	4 (7.9)

* SD - standard deviation.

## DISCUSSION

Deficiency of vitamin D, also known as 'sunshine vitamin', is a common health problem, very often unrecognized and untreated. Classically, vitamin D deficiency has been known to cause growth retardation, rickets, and dental caries in children, and osteomalacia, osteopenia, and decreased muscle strength in adults.^6^ Apart from role in calcium homeostasis, recent evidences support immunomodulatory effects of vitamin D in human body.^7^ Cardiovascular effects of vitamin D has been hypothesized with demonstration of vitamin D receptors in endothelial cells, lymphocytes, macrophages, cardiomyocytes, vascular smooth muscle cells, and B pancreatic cells. Low vitamin D levels upregulate renin angiotensin aldosterone system (RAAS) and cause endothelial dysfunction to promote atherogenesis.^8^ In a study by Dziedzic et al., lower 25(OH) vitamin D levels were found in patients with one to three vessel atherosclerosis as compared to those who did not have significant lesions in their coronary arteries.^9^

High prevalence of vitamin D deficiency has been reported in patients of coronary artery disease in studies conducted in different parts of the world. A study by Akin et al. from Korea concluded that patients with low 25(OH) vitamin D levels had higher odds of having significant coronary artery stenosis.^10^ Similarly, prevalence of vitamin D deficiency was reported to be 98.3% in patients of ACS in a study from New Delhi, India by Roy et al.^11^ Karur et al. concluded that the prevalence of vitamin D deficiency was 83.5% among patients of acute myocardial infarction in another study from India.^4^ In our study, the prevalence of vitamin D deficiency was 64.7% (51.58-77.82 at 95% CI) in patients of acute coronary syndrome. This study adds to the evidence regarding vitamin D status in our context.

In our study, the mean vitamin D levels were found to be lower in female patients and in patients who hailed from hilly region of Nepal (Table 3). Vitamin D insufficiency is known to be associated with indoor lifestyle, sun avoidance strategies, distance from the equator, darker skin and winter season.^7^ Gender wise significant difference could be related to the nature of indoor/outdoor activities as defined by classical gender roles of our study population. Similarly, the difference on basis of hills or terai could be related to the degree of sunlight exposure and seasonal variation in these areas.

On stratifying mean vitamin D levels to some prespecified cardiovascular risk factors, mean vitamin D levels were found to be lower in patients with diabetes mellitus, systemic hypertension, and dyslipidemia (Table 3). Similar kind of finding was also seen in the National health and nutritional examination survey done in United States, which showed that the mean 25(OH) vitamin D levels were lower in females, in those with history of hypertension, diabetes mellitus or dyslipidemia with P value <0.001.^12^ Observational studies have recently linked vitamin D levels to factors influencing cardiovascular health; though causal association is yet to be defined. Chronic vitamin D deficiency results in secondary hyperparathyroidism, which eventually has been linked to insulin resistance and development of diabetes mellitus and metabolic syndrome. In a study by Mathieu, et al. administration of 1,25(OH) vitamin D3, prevented the development of type 1 diabetes mellitus in animal models.^13^ Vaidya and Forman reported lower vitamin D levels to be associated to a higher blood pressure and a higher risk of developing systemic hypertension.^14^ In a study by Rejnmark, et al. plasma levels of triglyceride inversely correlated with seasonal change of vitamin D levels.^15^ This finding highlights the influence of vitamin D on plasma lipid profile and cardiovascular health.

Emerging evidence of vitamin D having anti-inflammatory, anti-proliferative, anti-diabetic, anti-hypertrophic and anti-thrombotic effects have intrigued researchers worldwide. While interpreting the findings from our study, it is necessary to consider the prevalence of vitamin D deficiency in general population. Studies from Indian subcontinent countries report prevalence of hypovitaminosis D to range from 70 to 100% in general population.^16^ Likewise, in a study by Poudel et al., 70% of total study population who attended outpatient clinic at a tertiary level hospital in Kathmandu, Nepal, had vitamin D deficiency, more commonly found among females and elderlies.^17^ Our study reports high prevalence (64.7%) of vitamin D deficiency in patients of acute coronary syndrome with lower mean vitamin D levels in patients with well-known cardiovascular risk factors like diabetes mellitus, hypertension and dyslipidemia. However, in view of high prevalence of vitamin D deficiency in general population, these findings need to be strengthened with adequately powered randomized controlled trials in future. Until then, possible association or causality can't be proved.

The main limitation of this study was small sample size. Larger well planned studies need to be done to prove causal association of vitamin D to different cardiovascular risk factors. Detailed data, related to diet and skin pigmentation of patients, were not collected, which could have influenced vitamin D levels in our patients.

## CONCLUSIONS

The prevalence of vitamin D deficiency, among patients of acute coronary syndrome in our study, was sufficiently high and comparable to various other homologous studies done in the past. The association of vitamin D deficiency to different cardiovascular risk factors must be tested in well powered trials in future to evaluate for causality. Vitamin D deficiency is treatable and supplementation is inexpensive. With the evolving non-skeletal health benefits of vitamin D, it could be potentially considered as an important prophylactic measure to prevent cardiovascular morbidity and mortality in high-risk patients.
